# Editorial: Methods in phytohormone detection and quantification: 2022

**DOI:** 10.3389/fpls.2023.1235688

**Published:** 2023-07-10

**Authors:** Da Cao, Man He

**Affiliations:** ^1^ Laboratory of Functional Plant Biology, Department of Biology, Ghent University, Ghent, Belgium; ^2^ Key Laboratory of Analytical Chemistry for Biology and Medicine (Ministry of Education), Department of Chemistry, Wuhan University, Wuhan, China

**Keywords:** plant hormone, quantification, detection, mass spectrometry, analytical chemistry, plant development

Despite their importance for plant growth and development, not all phytohormones have been found and their functions still remain largely unclear (Umehara et al., 2008). One of the important reasons for this is that it is difficult to detect the existence of some plant hormones such as brassinosteroids (BR) and strigolactones (SL), as they express at very low concentrations. As a result, progressive techniques for plant hormone detection and quantitation are needed to study their roles. This special Research Topic, “*Methods in phytohormone detection and quantification*” comprises five research articles through both original articles and reviews. It aims to highlight new and improved techniques for phytohormone detection and quantitation and provide updated approaches to study phytohormones and their roles in plant development and growth.

## Past methods

Auxin was the first identified phytohormone in history. To measure the concentration of auxin, the *Avena* Geo-curvature test was proved to be the most sensitive and accurate method in the 1950s (Kaldewey et al., 1968). After the identification of auxin, other phytohormone classes such as ethylene (ETH), cytokinin (CK), gibberellin (GA), abscisic acid (ABA) and salicylic acid (SA) were gradually found (Neljubow, 1901; Miller et al., 1956; Addicott and Lyon, 1969; Raskin, 1992). To discover the function of these phytohormones, it was necessary to quantify their concentrations in plant tissues. Hence, in the 1980s, immunoassay was chosen and widely applied to solve this problem (Weiler, 1982). Immunoassay measures phytohormone concentrations using the affinity between labeled antigens (phytohormones) and specific antibodies, but can only be applied to quantify the phytohormones with high concentrations since its sensitivity and specificity are low. Other approaches to detect and quantify phytohormones such as molecular imprinting (Kugimiya and Takeuchi, 1999), have been tried by some scientists, but that approach has rarely been applied in recent years.

## Current methods

Nowadays, chromatography coupled with mass spectrometry has become the most efficient and powerful method to identify and quantify phytohormones. Chromatographic techniques such as gas and liquid chromatography are available to separate compounds with different polarity, molecular weight and/or electronic charge. Mass spectrometry can identify what compounds they are *via* identifying their accurate molecular weights. With high resolution, chromatography coupled with mass spectrometry has been introduced to measure phytohormones for a long time. In 1969, gas chromatography-mass spectrometry (GC-MS) was first applied to measure the concentration of GA (Binks et al., 1969). With the development of this technique, tandem mass spectrometry (MS/MS) was invented, which provides more sensitivity and accuracy than the original mass spectrometry. The core function of MS/MS is to determine the mass to charge ratio (m/z) of compounds using a first mass analyzer followed by fragmentation of each compound in a collision cell with subsequent mass analysis of these fragments. For instance, Müller et al. (2002) established a GC-MS/MS method to detect and quantify five acidic phytohormones. In our selection, Chen et al. measured three classes of phytohormones, IAA, GA and ABA using GC-MS/MS. However, GC-MS/MS is limited to the analysis of volatile and thermostable phytohormones (Chiwocha et al., 2003). Hence, more and more scientists have recently started using high-performance liquid chromatography coupled with tandem mass spectrometry (HPLC-MS/MS) to measure phytohormones (Ljung et al., 2010; Walton et al., 2015). To date, HPLC-MS/MS has been applied for the quantification of various phytohormones such as auxin, ABA, CKs and SLs simultaneously (Chiwocha et al., 2003; Pan et al., 2010; Cao et al., 2017; Šimura et al., 2018; Xin et al., 2020).

In this Research Topic, Cao et al. developed and validated an HPLC-MS/MS method for quantifying four classes of shoot branching-related phytohormones in small pea axillary buds (around 10 mg). Remarkably, this method enables the extraction of phytohormones and nucleic acids from the same sample, which significantly facilitates the comparative analyses of phytohormones and gene expression. Later on, this method was applied to monitor phytohormone level changes in axillary buds after decapitation. Combined with plant physiological research, their dataset helps to provide a new model of how phytohormones control axillary buds to progress from a state of arrested growth into branches (Cao et al., 2023).

A second protocol, Zhao et al., developed a novel approach that combines a HPLC-MS/MS method and a tobacco syringe agroinfiltration assay to test phytohormone transporter activity. Using the endogenous hormones in tobacco leaves as the substrates, this method was validated by successfully detecting the activity of three known hormone-exporting transporters for ABA, JA and CK. It also identified an unknown CK exporting transporter from maize. This established method provides a rapid approach for evaluating the activity of transporter candidates or for the screening of new phytohormone transporters. However, as the authors stated, their method cannot provide the same level of sensitivity or kinetic data on phytohormone transport compared with isotope label-based methods.

## Outlook

Phytohormones play important roles in coping with non-optimal environmental conditions and regulating plant development. In this selection, Li et al. and Li et al. reviewed the phytohormone roles in regulating trichrome and root hair development. Phytohormone interactions and related gene networks are also summarized in both of the papers. As discussed, to elaborate on the roles of phytohormones, It is important to collect and integrate datasets generated from different omics technologies such as proteomics and transcriptomics with hormone profiling results. For example, Chen et al. performed transcriptomic analysis and hormone profiling together and provided solid information about phytohormone-related metabolic processes and genes involved in the salt resistance of *Sesuvium portulacastrum*.

To date, there are still phytohormones that cannot be easily detected or quantified. For example, phosphate deficiency which boosts endogenous SL production is required for SL quantification in most plant species due to the hardly detectable levels of SL under normal growth conditions (Umehara et al., 2008; Boutet-Mercey et al., 2018; Rial et al., 2019; Floková et al., 2020). However, this is not applicable to some studies which require sufficient phosphate nutrients in the soil. Thus, new methods or techniques which provide higher sensitivity and selectivity are still urgently needed for phytohormone research.

## Author contributions

DC drafted the manuscript and MH critically reviewed it. All authors contributed to the article and approved the submitted version.
